# Music performance anxiety: Insights from psychological science

**DOI:** 10.3389/fpsyt.2025.1675660

**Published:** 2025-10-24

**Authors:** Stephen Lim, Narongrit Dhamabutra

**Affiliations:** ^1^ Department of Western Music, Faculty of Fine and Applied Arts, Chulalongkorn University, Bangkok, Thailand; ^2^ Department of Educational Research and Psychology, Faculty of Education, Chulalongkorn University, Bangkok, Thailand

**Keywords:** music performance anxiety, performance science, performance practice, psychological science, music cognition, music education

## Introduction

Music performance anxiety (MPA) commonly refers to the experience of marked and persistent anxious apprehension related to musical performance that has arisen through specific anxiety-conditioning experiences (1). Studies estimating the prevalence of MPA among music students and professionals have reported wide-ranging figures from 16% to even 96% (for recent reviews, see 2–4). Numerous coping strategies and interventions have been developed over the years (for reviews, see 3–9), although MPA prevalence rates have not substantially changed at the population level over the years, suggesting that extant interventions on overall may have limited impact (4). Importantly, treating the symptoms of MPA does not necessarily resolve its root cause. Musicians have reported experiencing anxiety far in advance of their performance, rather than only during their actual performance on stage (10). In reality, music performance in evaluative situations carries high demands—whether perceived or actual—on the performer to attain (near) perfection. Such pressures and strivings for errorless performance and perfection may be particularly acute in some genres such as Western classical music, especially in solo or soloist performances (11, 12; see also 13), where musicians face challenges ranging from temporally precise fine motor technique, sustaining attention over long durations, memorizing complex material, to interpretative and musical communication skills (14, 15). Yet, many MPA studies do not screen participants for weak technical musical ability, and for whom anxiety may be a *consequence* (rather than a cause) of poor performance (7). As Faur et al. (5) note in their review, that extant MPA interventions do not always improve performance quality could be due to their focus on treating anxiety symptoms without targeting the improvement of technical skill (see also 16). Indeed, debilitating anxiety could result from inadequate preparation and deficits in musical technique.

## Psychological insights

Do test-anxious persons actually perform worse in evaluative situations than their knowledge or skill would otherwise allow? This commonly held but rarely tested assumption has recently been challenged in a field study. Using log files from a digital learning platform, Theobald et al. (17) examined data from 309 medical students preparing for a high-stakes exam. The students’ test anxiety at the start of the study was negatively associated with their final exam performance, as well as their preparation performance when completing old exam questions over 100 days before their final exam, and their performance on a mock exam 28 days before their final exam. If students’ test anxiety (e.g., fears about knowledge deficits or negative consequences of poor exam performance) interferes with their processing and performance during evaluative situations, then high-anxiety students should perform worse on the high-stakes final exam than non-evaluative mock exam, and this performance decline should correlate with their test anxiety. But this was not the case. Instead, students’ test anxiety did not predict a drop in their performance from the mock exam to final exam. Furthermore, when controlling for students’ mock exam performance, their test anxiety did not predict their final exam performance.

These findings reveal that highly test-anxious students’ lower knowledge levels accounts for the link between their test anxiety and worse final exam performance. In line with this idea, Theobald et al. (17) found that students with high test anxiety tended to show less knowledge gains during their exam preparation. Moreover, whereas higher state anxiety in the morning did not predict the number of practice questions that students correctly solved on that day during their exam preparation, correctly solving fewer questions predicted students’ higher state anxiety the next day. Thus, awareness of one’s poorer knowledge may trigger test anxiety. The key implication is that short-term interventions which aim to reduce test anxiety shortly before or during the evaluative situation may have limited impact because they cannot offset long-term knowledge deficits. Rather, effective learning strategies that boost and secure actual deep learning are needed to tackle test anxiety at its root.

These findings have important implications for music performance practice. Some recent evidence suggests that MPA and performance quality co-develop during practice sessions, such that musicians who improve their playing are subsequently less anxious (18). Thus, MPA interventions ought to promote effective practice that produces optimal, deep learning at an early stage during performance preparation, and not merely aim to reduce anxiety only shortly before or during stage performance.

Music students have most commonly reported that increasing practice time is their main strategy to manage MPA, while developing specific practice techniques to address concerns such as potential memory slips (10) and difficult parts of the music (19). Notably, students have ascribed their performance experiences to their level of technical preparedness, which may directly impact their MPA. For instance, interviewed participants in Tahirbegi’s (10) study commented that “My anxiety depends on how secure I feel about the concerts, how well I know the pieces I play” and “I notice that if I am very well prepared, I don’t necessarily get that nervous”. Indeed, well-prepared performers may experience higher self-efficacy that facilitates their management of MPA. In turn, lower MPA mediates the positive effects of self-efficacy on self-rated performance quality (20). Conversely, more anxious musicians have reported less conviction in their ability to perform the task (i.e., lower self-efficacy) even with their anxiety under control than less anxious musicians (21). Thus, mastering the musical material and technical skills could boost self-efficacy, which has been found to be the most important predictor of music performance quality in evaluative exams (22).

Clearly, musicians are well-aware of the importance of good preparation for successful musical performance (23). However, the practice strategies that they use during their preparation may not always be the most effective. For instance, although increasing practice time is a strategy that musicians often use to cope with MPA (10) and that has been associated with lower self-reported MPA in some studies (e.g., 20, 24, 25), hours of practice alone do not necessarily predict better music performance quality (26, 27). Moreover, state anxiety has been associated with repetitive practice behaviors during longer practice sessions, which may increase the musician’s risk of developing injuries from overuse and repetitive strains (18). Adding insult to injury, mindless repetition and drill during practice may also have limited benefits for deep learning and performance quality (23, 28). Taken together, these findings point to the importance of practice *quality* for achieving musical mastery and managing MPA. How, then, should we practice for deep, durable learning?

## Learning strategy #1: Deliberate practice

One potential strategy is *deliberate practice*, which involves structured, effortful practice activities that are specifically designed and tailored to improve one’s current level of performance, as characterized by: (a) repeated experiences in which the performer attends to the critical task elements, (b) focused attention, (c) intrinsic motivation to improve, and (d) corrective feedback and performance monitoring that enable gradual refinements (23). Many studies have since shown that deliberate practice is a necessary and important predictor of higher levels of music performance (e.g., 23, 29, 30; see 31 for a review), even if it may not be sufficient (32, 33). For instance, Platz et al.'s (34) meta-analysis of 13 studies found a mean effect size of *r* = 0.61 accounting for 36% of the variance in the relationship between task-relevant practice (including deliberate practice) and music performance (see also 32, 35, 36). Indeed, practice time has been shown to predict musical achievement only through formal practice that includes deliberate practice and self-regulation strategies, goal direction, and focused attention—when formal practice is controlled for, then practice time in fact negatively predicts musical achievement (37). This suggests that informal practice time, which includes improvisation, playing one’s favorite pieces by ear, and messing about with music, may not only be “empty” time, but could impede musical achievement.

## Learning strategy #2: Retrieval practice

Another potential strategy is *retrieval practice*, a potent learning technique that decades of research have robustly shown fosters deep, durable learning (38, 39; see 40 for a review). Notably, retrieval practice yields reliable memories that are resilient to stress. In a study by Smith et al. (41), participants studied words and images either by restudying them or practicing retrieval (i.e., recalling as many items as they could after studying them). One day later, half of the participants underwent stress induction in which they were asked to give impromptu speeches and solve math problems in front of an audience. When participants were subsequently tested on their memory of the studied words and images, the retrieval practice group outperformed the restudying group. Importantly, stress impaired memory in the restudying group, but not in the retrieval practice group. In fact, stressed retrieval practice participants outperformed non-stressed restudying participants, and performed just as well as non-stressed retrieval practice participants. These findings suggest that stress does not always harm memory and performance. Rather, when strong memories are created during encoding by using effective techniques such as retrieval practice, these well-encoded memories are protected against stress. At the same time, retrieval practice reduces test anxiety. In a study of 1,408 middle and high school students, Agarwal et al. (42) found that 72% of students reported feeling less nervous for tests and exams for their classes that had implemented retrieval practice (e.g., via low-stakes practice quizzes administered via “clickers”). Presumably, when students engaged in retrieval practice, they learned the course material better and were thus less anxious about facing subsequent evaluative tests. In the context of music performance, practicing retrieval during performance preparation could be a powerful way to lower MPA, while enhancing learning and reducing memory failures during one’s actual performance even when anxious.

## Barriers to implementing effective learning strategies

Although deliberate practice and retrieval practice are promising techniques for enhancing deep learning and managing MPA, their benefits may be constrained by suboptimal implementation. For instance, rote memorization (e.g., repeating the music until it can be played automatically by “feel”) is a common form of retrieval practice, especially for novice musicians (43, 44; see 45 for a review of memorization methods). After repeating short sections and playing them from memory without the music score, the musician may link or “chunk” these sections into longer sections, then repeat these longer sections and play them from memory, until the whole piece has presumably been memorized. In this way, retrieving each chunk cues the musician’s recall of the next chunk (46), in what is known as associative chaining (47) or serial position effects in recall (e.g., 48).

Yet, although some degree of automaticity is needed for efficient performance under pressure, procedural memory is unreliable and vulnerable to interference (44, 45, 49, 50). When memory fails during a music performance, serial cueing of recall is disrupted (51). Thus, if musicians relied only on rote memorization during their performance preparation, they may have no other recourse than muddling along, improvising or, worse still, restarting the piece if a memory lapse occurred on stage (46; see also 52).

In fact, expert performance has been conceptualized as a shift of cognitive control to higher-level strategic aspects of performance, rather than being eliminated entirely for full automaticity (53). That is, experts may be able to perform seemingly without thinking because they harness automatic control systems to think in music while sequencing the actions needed (54). For instance, expert musicians form performance cues based on the musical structure of a piece (55) to create memory that is “content addressable” (52). Such performance cues include: (a) *structural cues* representing section boundaries of the music (e.g., based on harmonic and melodic properties), (b) *expressive cues* representing the feelings to be conveyed to an audience, (c) *interpretive cues* representing critical interpretive decisions that need monitoring during the performance (e.g., decreasing the dynamics to prepare for a later crescendo), and (d) *basic cues* representing critical technique details such as fingering (55). In this way, the music’s structure provides a hierarchical retrieval scheme, while performance cues serve as landmarks in a mental map from which the musician can access specific content or locations to recover from a memory lapse, rather than having to restart from the beginning of the piece.

But even performance cues have limitations. Although the musician may be able to recover from a memory lapse by jumping forward and continuing from a performance cue, recall tends to be highest at the beginning of phrases starting with structural or expressive cues, then declines steadily across the rest of the phrase (50, 56). One interpretation is that structural and expressive cues provide content-addressable access to memory that is otherwise organized as an action sequence (i.e., in serial order). Thus, later bars in the sequence are recalled less well and memory failure becomes increasingly likely. In contrast, recall tends to be lower at basic cues then increases as serial distance from the cue increases, suggesting that basic cues do not provide direct content-addressable memory access but function as parts of serial associative chains. How, then, can we bolster content-addressable memory access?

## Learning strategy #3: Non-serial practice

**Figure 1:**
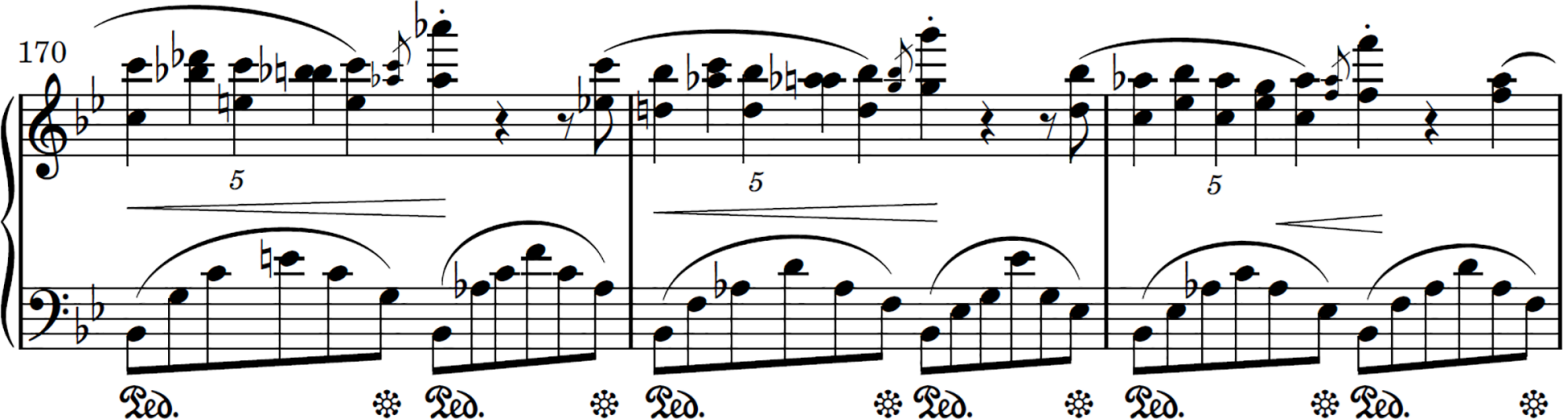
Excerpt from Chopin’s Ballade no. 1 in G minor, op. 23.

Through personal performance practice and experience, we observed the prowess of *non-serial* learning and practicing in breaking serial associative chains. Consider the excerpt from Chopin’s (57) first ballade (**Figure 1**). It is commonplace for bars 170 to 172 to form a serial associative chain so that, relative to bar 170, the content of bar 171 or 172 is less readily accessible. The retrievability of bar 171 often hinges heavily, if not wholly, on first executing bar 170 successfully; likewise, bar 172 relies on bar 171. Evidently, where a pianist fails to execute bar 170 successfully, bar 171 and, by extension, bar 172 also cannot be recovered. Indeed, such is the severe cost to basic cognitive systems that function in a serial recall manner. Consequently, we noticed one cannot successfully start performing a piece from just anywhere, but must do so from the piece’s outset or at best the beginning of a section. For a singer, one often cannot directly recall what words in a ballad rhyme with *ee* or even what happens in the last stanza, but has to in fact sing the ballad, listen for *ee* sounds and pay attention to the last stanza when one gets to it (52). Similarly, we observed that a pianist in performance often cannot directly pinpoint the exact notes that particular bars contain, and would have to “play to know” when one gets to each of those bars. Under this light, one ought to not practice bars 170 to 172 of Chopin’s ballade categorically. Toward dissolving serial associate chains, a pianist could, for instance, subdivide each bar into smaller halves and begin practicing each bar from its midpoint—from bar 170b to 171a; from bar 171b to 172a, thereby strengthening content-addressable memory access for optimizing stage performance. We encourage empirical investigations in systematically assessing the efficacy of non-serial practice, and welcome collaborations.

## Conclusion

Music performance anxiety (MPA) negatively impacts musicians in almost every musical context imaginable. Yet, extant interventions do not remedy MPA at its core. Drawing on psychological science, performance-anxious musicians should not perform worse in high-stakes stressful situations than their preparation (practice) would otherwise allow. Instead, where musicians are well-prepared, performance anxiety per se should not interfere with stage performance accuracy and quality. Thus, rather than aim to reduce anxiety only shortly before or during stage performance, performance-anxiety interventions ought to promote effective (e.g., non-serial) practice and optimal learning at an early stage during performance preparation. We hope this paper will spur further dialogue about new pathways toward deeper musical learning and fuller onstage flourishing.
